# Determination of Residual Diisocyanates and Related Diamines in Biodegradable Mulch Films Using *N*-Ethoxycarbonylation Derivatization and GC-MS

**DOI:** 10.3390/molecules27196754

**Published:** 2022-10-10

**Authors:** Kai Cai, Yechun Lin, Yunfei Ma, Zhixiao Yang, Lei Yu, Jie Zhang, Dongqing Xu, Rong Zeng, Weichang Gao

**Affiliations:** 1 Guizhou Academy of Tobacco Science, Upland Flue-Cured Tobacco Quality & Ecology Key Laboratory of CNTC, Guiyang 550081, China; 2 Key Laboratory for Degradation Technologies of Pesticide Residues with Superior Agricultural Products in Guizhou Ecological Environment, Guiyang University, Guiyang 550005, China; 3 School of Geography Science, Nanjing University of Information Science and Technology, Nanjing 210044, China

**Keywords:** diisocyanates and related diamines, ultrasonic hydrolysis and extraction, *N*-ethoxycarbonylation, GC-MS, biodegradable mulch film

## Abstract

Diisocyanates are highly reactive compounds with two functional isocyanate groups. The exposure of diisocyanates is associated with severely adverse health effects, such as asthma, inflammation in the respiratory tract, and cancer. The hydrolysis product from diisocyanates to related diamines is also a potential carcinogen. Here, we developed an effective, accurate, and precise method for simultaneous determination of residual diisocyanates and related diamines in biodegradable mulch films, based on *N*-ethoxycarbonylation derivatization and gas chromatography-mass spectrometry. The method development included the optimization of ultrasonic hydrolysis and extraction, screening of *N*-ethoxycarbonylation conditions with ethyl chloroformate, evaluation of the diamines degradation, and analysis of the fragmentation mechanisms. Under the optimum experimental conditions, good linearity was observed with *R*^2^ > 0.999. The extraction recoveries were found in the range of 93.9–101.2% with repeatabilities and reproducibilities in 0.89–8.12% and 2.12–10.56%, respectively. The limits of detection ranged from 0.0025 to 0.057 µg/mL. The developed method was applied to commercial polybutylene adipate co-terephthalate (PBAT) biodegradable mulch film samples for analysis of the diverse residual diisocyanates and related diamine additives. The components varied greatly among the sample from different origin. Overall, this study provides a reliable method for assessing safety in biodegradable mulch films.

## 1. Introduction

Plastic mulches are widespread in the world and have a profound impact in agriculture [1]. Plastic mulches can retain humidity and heat, prevent soil, erosion and weed development, and then regulate microclimate for crop growth, which can effectively increase the yield by 20–50% [2]. However, due to their stability and high durability, traditional plastic films with polyethylene (PE), polyvinyl chloride (PVC) etc. have long degradation time and easily accumulate with micro-plastic (plastic particles < 5 mm) in different environments, which will affect the health of wildlife and human [3]. In recent years, biodegradable mulch films have been proposed as an alternative way to solve the white pollution, and polybutylene adipate co-terephthalate (PBAT) is one of the most promising biodegradable materials. Due to its excellent flexibility, high strength, and good tear resistance, it is widely used in the production of mulch and package [4].

In order to improve the performance of the mechanical properties in PBAT, the diisocyanates are widely used as an additive. The modified PBAT polymer is formed by chemical reaction between a diisocyanate and a diol or polyol in the presence of a catalyst and other additives [5]. However, there are still some residual diisocyanates unreacted and attached to the polymer [6]. These diisocyanates are a family of highly reactive and relatively low molecular weight compounds with two isocyanate functional groups (−NCO). The impacts of PBAT on environmental and health are more related to residual diisocyanate components rather than their polymer, due to the monomer capability to interact with membrane cells [7]. As we all know, the high chemical reactivity of diisocyanates makes them toxic compounds and their adverse health effects are well-documented [8]. Overexposure in diisocyanates can lead to allergic respiratory diseases, such as occupational asthma, hypersensitivity pneumonitis, or alveolitis [9]. These compounds can also cause skin and eye irritation [10]. In addition, diisocyanates act as electrophilic agents and can react with DNAs to produce genetic damage and then are classified as group 2B (possibly carcinogenic to humans) by the International Agency for Research on Cancer (IARC) [11]. Therefore, the production of diisocyanates and products containing diisocyanates are strictly regulated from the point of view of health and safety at work. The biological guidance value (BGV) has been established for monitoring exposure levels in German Research Foundation (DFG) and the American Conference of Governmental Industrial Hygienists (ACGIH). The hydrolysis products from diisocyanates to related diamines were also a potential carcinogen [12]. Therefore, quantifying residual diisocyanates and related diamines levels in PBAT biodegradable mulch films are necessary to ensure safety.

Cui et al. developed a microwave-assisted extraction and dispersive liquid-liquid microextraction with gas chromatography-mass spectrometry (GC-MS) for the determination of eighty organic additives in biodegradable mulch films, including several diisocyanates [13]. Diisocyanates can react with protonated solvent, and this method showed poor feasibility for analyzing diisocyanates with poor linearity and extraction recovery. In order to accurately analyze diisocyanates, the diisocyanates must be hydrolyzed with acid media to release free diamines and then detect the total amounts of diamines [10]. The most common hydrolysis methods involve an HCl or H_2_SO_4_ solution for overnight reaction with a high temperature in biological matrix, such as urine and blood [14,15,16]. These methods need a long time for hydrolysis and are limited to one or two classes of diisocyanate [17]. However, diverse diisocyanate additives including aliphatic (e.g., hexamethylene diisocyanate), alicyclic (e.g., isophorone diisocyanate), and aromatic diamine (e.g., toluene diisocyanate) have been applied in traditional plastic and biodegradable material products [18]. Different classes of diisocyanates have different hydrolysis properties [10], it is important to screen suitable hydrolysis conditions for diverse diisocyanates in PBAT biodegradable mulch films.

A number of separation techniques have been developed to detect aliphatic diamines and aromatic diamines, which include capillary gas chromatography, high performance liquid chromatography (HPLC), capillary zone electrophoresis (CZE), and ion-exchange chromatography (IC) [9,19,20,21]. GC often provide best separation capabilities with highest theoretical plates and minimal matrix effects on peak resolution, which make it most suitable for analysis of diverse diamines [22]. However, direct analysis of diamines by GC is difficult due to their high polarity and poor volatility [23]. Hence, a derivatization reaction is mandatory. Ethyl chloroformate (ECF) has been shown to be a rapid and strong derivatizing reagent [24]. Compared with silylating derivatization, chloroformate derivatization can be performed within one minute at room temperature directly in the aqueous medium [25]. Furthermore, ECF derivatization can balance steric hindrance for reaction and water solubility for derivative products in contrast to other alkylchloroformate reagents [26]. The diamines can be derivatized with ECF under basic aqueous conditions, and the *N*-ethoxycarbonylation derivatives are more lipophilic than the parent diamines, facilitating extraction with organic solvent and separation with GC [27]. As far as we know, ECF derivatization followed by GC-MS analysis has not previously been used to analyze diverse diamines in PBAT biodegradable mulch films.

In the present study, we developed a new method for the determination of diverse residual diisocyanates and related diamines with simultaneous ultrasonic hydrolysis and extraction, *N*-ethoxycarbonylation using ECF and then followed by GC-MS analysis. In order to obtain the effective, accurate, and precise requirement, ultrasonic acid hydrolysis extraction was optimized with Box-Behnken Design (BBD) and degradation of diamines in aqueous phase under alkaline conditions were evaluated under *N*-ethoxycarbonylation conditions. The fragmentation behaviors of *N*-ethoxycarbonylation derivatives were confirmed and could potentially be used for screening and identification of potential diisocyanates and related diamines additives. The developed method was employed on determination of residual diisocyanates and related diamines in PBAT biodegradable film samples with different manufacturers and the components can be used to assess the safety.

## 2. Results and Discussion

### 2.1. Optimization of Ultrasonic Hydrolysis and Extraction

**Figure 1:**
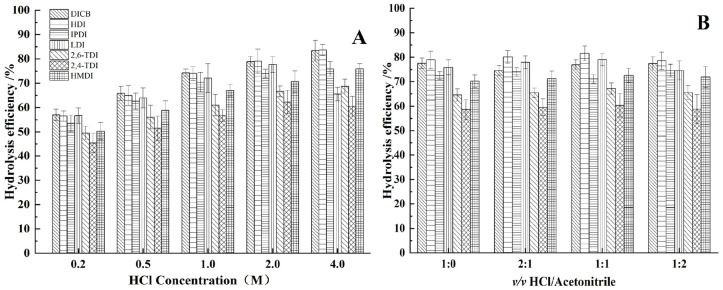
Effect of HCl concentration (**A**) and the ratio of *v/v* HCl/acetonitrile (**B**) on hydrolysis efficiency from diisocyanates to diamines.

The HCl has been used for hydrolysis from diisocyanates to diamines [28]. Considering that diisocyanates hydrolysis and diamines extraction were carried out simultaneously, a certain amount of water-soluble organic solvent was required to improve the extraction ability. Methanol can react with diisocyanates to form methyl carbamates, whereas acetone can react with aromatic diamines to form Schiff bases at acid conditions [13,29]. Hence, acetonitrile was applied in this study. Hydrolysis efficiency was evaluated using diisocyanates with different conditions. The HCl concentration varied from 0.2 to 4 M. Figure 1A showed the hydrolysis efficiency increased with the increasing HCl concentration, but LDI decreased at 4 M HCl. This may be due to hydrolysis of β-aminocarboxylic esters in LEE to L-lysine in high concentration acid. The *v/v* ratio of HCl and acetonitrile varied from 1:0 to 1:2. Figure 1B showed the acetonitrile had little effect on hydrolysis efficiency, but high ratio of acetonitrile affected drying of the derivative product. Hence, 2M HCl/acetonitrile (*v/v* = 1:1) was selected for further optimization.

To further improve hydrolysis efficiency, a multivariate strategy based on the response surface methodology with a four-factor, three-level BBD was further employed to optimize important ultrasonic acid hydrolysis conditions, such as liquid-to-material ratio/*v/w* (A), ultrasonic intensity power/W (B), hydrolysis temperature/°C (C), and time/min (D). BBD is a class of rotatable or nearly rotatable second-order designs based on combinations of three levels [30]. The advantage is that it is more efficient, convenient, and less expensive with no axial points [31]. The BBD of coded variables, uncoded variables, and average hydrolysis efficiency were presented in Table 1. The coded variables were set as −1, 0, and +1, and corresponded with the uncoded variables of A (−1 = 40, 0 = 60, +1 = 80 mL/g), B (−1 = 100, 0 = 200, +1 = 300 W) C (−1 = 30, 0 = 50, +1 = 70 °C), and D (−1 = 30, 0 = 60, +1 = 90 min). Due to the same change trend, the responses were evaluated with average hydrolysis efficiency of each class diisocyanates (aliphatic, cycloaliphatic, amino acid ester and aromatic). To evaluate how well the model fits the experimental results, the BBD was evaluated at a 5% level of significance and validated using analysis of variance (ANOVA) in Table 1. The result showed that model of each class diisocyanates was highly significant, with a *p*-value < 0.0001. The coefficient of determination (*R*^2^) was greater than 0.9018. A lack of fit *p*-value greater than 0.05 indicated that the lack of fit was not significantly associated with the pure error. Additionally, smaller coefficient of variation (CV) (less than 3.61%) indicated that each model was reproducible. These statistic parameters demonstrated the BBD model reliability.

To further predict the optimum conditions, the model variables were set according to our specific requirements. The liquid-to-material ratio showed a statistically non-significant effect. Considering saving solvent and obtaining high hydrolysis efficiency, the goals for variable of A were set as 60 *v/w*. B, C, and D were set as ‘‘in investigated range” (from –1 to +1 level). Because the maximum hydrolysis efficiency is our aim, the goal was set to ‘‘maximize”. Then, the solutions were obtained with Design Expert software. The result showed that the highest hydrolysis efficiency was 94.0% for aliphatic diisocyanates, 94.4% for cycloaliphatic diisocyanates, 86.8% for amino acid ester, and 81.3% for aromatic diisocyanates and its optimum conditions were 101.7 W for ultrasonic intensity power, 30.2 °C for hydrolysis temperature, and 75.9 min for time. Low-intensity ultrasonication is believed to generate an unfocused ultrasonic beam and then facilitate the acid hydrolysis process under a fairly mild cavitation [32]. Hydrolysis temperature only had a statistically significant effect for amino acid ester. This may be attributed to higher temperatures easily resulting in hydrolysis of the ethyl ester bond. Further, the values were rounded off for simplicity of operation, and the optimum ultrasonic acid hydrolysis conditions were 100 W, 30 °C and 76 min. Three experiments were performed to validate this result. The average hydrolysis efficiency reached at 87.8%. This result showed that BBD model was adequate for reflecting the expected optimization.

### 2.2. Optimization of N-Ethoxycarbonylation Derivatization

Four alkylchloroformates (MCF, ECF, PCF and IBCF) were examined for the derivatization of different classes of diamines to provide a better chromatographic and characteristic fragmentation behaviors. The results showed that peak area decreased successively from MCF to IBCF, especially the 2, 4-TDA and 2, 6-TDA, perhaps due to the derivatization with larger steric hindrance in PCF and IBCF. However, characteristic ions became more and more obvious from MCF to IBCF, especially the aliphatic diamines. To balance qualitative and quantitative ability, ECF was chosen for derivatization.

**Figure 2:**
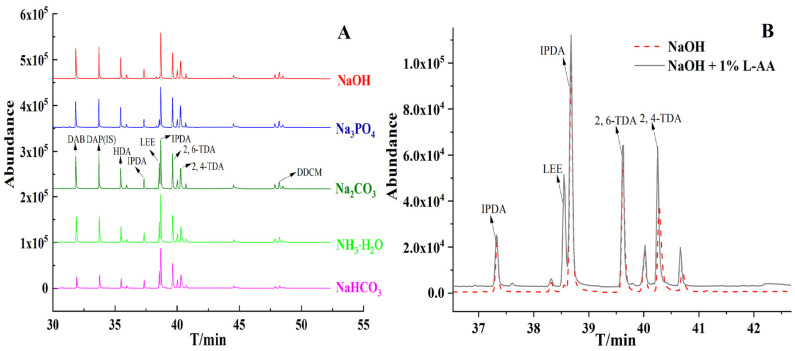
(**A**): Comparison of *N*-ethoxycarbonylation efficiencies in the five alkali with different pH (NaHCO_3_; pH = 8, NH_3_·H_2_O; pH = 9, Na_2_CO_3_; pH = 11, Na_3_PO_4_; pH = 12, NaOH; pH = 14); (**B**): Verification of degradation mechanism of LEE with addition of 1% (*w/v*) *L*-AA in NaOH (pH = 14).

Derivatization with ECF often needs alkaline aqueous media to convert diamines to *N*-ethoxycarbonylation derivatives. In order to achieve the purpose of diamine deprotonation, strong alkali is essential for this reaction. However, amines with certain structure are unstable under alkaline conditions and instability can accelerate with increasing alkalinity [33]. According to the pK_a_ of diamines within 7.33–10.87 in Table 2, we chose NaHCO_3_ (pH = 8), NH_3_·H_2_O (pH = 9), Na_2_CO_3_ (pH = 11), Na_3_PO_4_ (pH = 12) and NaOH (pH = 14) to adjust the pH for derivatization. Figure 2A reveals that the peak area of most diamines increased with increasing pH. However, Na_3_PO_4_ and NaOH significantly reduced the peak area of the LEE, particularly when NaOH was used, LEE was completely disappeared. This phenomenon is due to accelerating degradation with increasing pH. Previous studies have demonstrated that *β*-aminoketone are rapidly oxidative deaminate under stronger alkaline solutions [34]. LEE had similar structural units. Aromatic diamines also showed slightly lower peak areas at NaOH condition, which is attributed that aromatic diamines easily degraded to quinones with oxidation, with rates *p* > *m* > *o* isomer [35]. Therefore, for the analysis diverse diamines, Na_2_CO_3_ is more suitable for derivatization.

The structural units of LEE were *β*-aminocarboxylic ester, which is some different from the *β*-aminoketone of methcathinone analogues [33]. To verify whether *β*-aminocarboxylic ester is oxidized or not, *L*-AA was used to estimate the involvement of any oxidants in NaOH condition, such as dissolved oxygen or reactive oxygen species [36]. *L*-AA was added to the standard solution to give a final concentration of 1% (*w/v*). Subsequently, the pH was adjusted to 14 with NaOH. As shown in Figure 2B, it is obvious that the peak of LEE increased significantly with addition of *L*-AA. The aromatic diamines also had slightly increased. Theis phenomenon confirmed that *β*-aminocarboxylic esters were prone to oxidative degradation in strong basic media, which can be suppressed with addition of appropriate antioxidants.

Other parameters (ECF volumes, derivatization-extraction time, extraction solvent type, and extraction times) were further optimized to obtain the maximum derivatization efficiency. Insufficient ECF leads to incomplete derivatization, and excess ECF generates the HCl by-products to affect pH. The effect of ECF volumes was evaluated at 10, 30, 50, 70, and 90 μL in Table S1. For all targeted analytes, peak area was rapidly increased from 10 to 30 μL and kept constant at 70 μL and then slightly decreased at 90 μL. The derivatization-extraction time was taken into consideration and varied from 1–5 min, the results in Table S2 showed that the peak area remained unchanged. The *N*-ethoxycarbonylation derivatization is often fast. Besides, the extraction efficiency is related to the extraction solvent due to the difference in the properties of *N*-ethoxycarbonylation derivatives, different polarity solvent including petroleum ether, n-hexane, methyl tert-butyl ether, ethyl ether, ethyl acetate, and chloroform were evaluated with liquid-liquid extraction. The results in Table S3 showed that peak areas increased with increasing solvent polarity, and ethyl acetate and chloroform had highest extraction efficiency. Finally, the number of extractions was also evaluated, and third extraction repetition was found not to increase peak area. Considering more environmentally friendly, simplified operation, and maximum extraction efficiency, ECF volumes (50 μL), derivatization-extraction time (1–2 min), extraction (two times) with ethyl acetate were selected for simultaneous derivatization and extraction.

### 2.3. Mass Spectra Identification of N-Ethoxycarbonylation Derivatives

**Figure 3:**
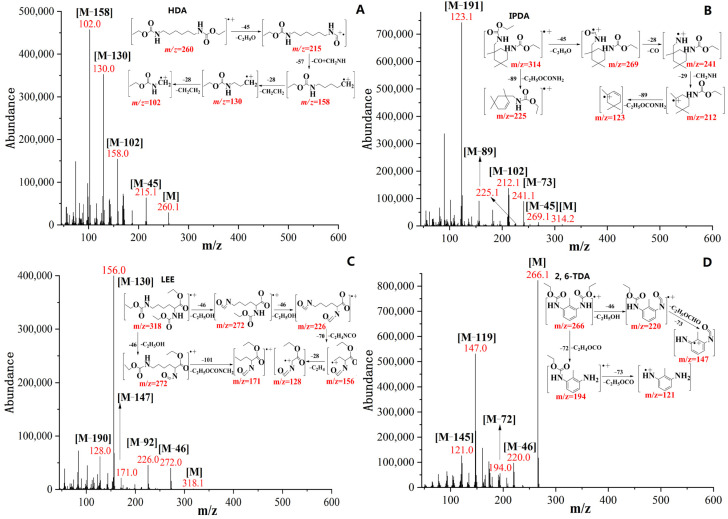
The main characteristic fragmentation in the *N*-ethoxycarbonylation derivatives of HDA (**A**), IPDA (**B**), LEE (**C**) and 2, 6-TDA (**D**).

The derivatization with ECF will result in the enhancement of molecular weight of analytes (+144) which leads to the formation of *N*-ethoxycarbonylation derivatives with decreased polarity and increased volatility. This allows improved chromatographic selectivity, reduced peak shape tailing, and shortened retention time with relatively better chromatographic behavior [37]. Generally, *N*-ethoxycarbonylation derivatives provided abundant characteristic fragment ions with a weak molecular ion in most cases, however, they were high molecular ion for aromatic diamines. The mainly characteristic ions were shown in Table 2. For the *N*-ethoxycarbonylation derivative of aliphatic diamines (HDA), Figure 3A showed a weak molecular ion peak at *m/z* 260. The main characteristic ions were *m/z* = 215, 158, and 130, which sequentially derived from a loss of *m/z* = 45 (-[C_2_H_5_O]), 57 (-[CO+CH_2_NH]) and 28(-[CH_2_CH_2_]) from molecular ion. The base peak ion with *m/z* =102 was due to the loss of *m/z* = 28 (-[CH_2_CH_2_]) from *m/z* = 130. For the *N*-ethoxycarbonylation derivative of cycloaliphatic diamines (IPDA), Figure 3B showed a weak molecular ion peak at *m/z* 314. The first fragmentation pathway was *m/z* = 269, 241 and 212, which sequentially derived from a loss of *m/z* = 45 (-[C_2_H_5_O]), 28 (-[CO]), and 29 (-[CH_2_=NH]) from molecular ion and the second fragmentation pathway was *m/z* = 225, which sequentially derived from a loss of *m/z* = 89(-[C_2_H_5_OCONH_2_]). The base peak ion with *m/z* = 123 was due to the loss of *m/z* = 89 (-[C_2_H_5_OCONH_2_]) from *m/z* = 212. For the *N*-ethoxycarbonylation derivative of amino acid ester (LEE), Figure 3C showed a weak molecular ion peak at m/z 318. The first fragmentation pathway was *m/z* = 272, 226 and 128, which sequentially derived from a loss of *m/z* = 46 (-[C_2_H_5_OH]), 46 (-[C_2_H_5_OH]) and 98 (-[CH_2_CH_2_+CH_2_CH_2_NCO]) from molecular ion and the second fragmentation pathway was *m/z* = 272 and 171, which sequentially derived from a loss of *m/z* = 46 (-[C_2_H_5_OH]) and 101(-[C_2_H_5_OCONCH_2_]). The base peak ion with *m/z* =156 was due to the loss of *m/z* = 70 (-[CH_2_CH_2_NCO]) from *m/z* = 226. For the *N*-ethoxycarbonylation derivative of aromatic diamines (2, 6-TDA), Figure 3D showed a strong molecular ion peak at *m/z* 266, which is also at base peak. The first fragmentation pathway was *m/z* =220 and 147, which sequentially derived from a loss of *m/z* = 46 (-[C_2_H_5_OH]) and 73 (-[C_2_H_5_OCO]) from molecular ion and the second fragmentation pathway was *m/z* = 194 and 121, which sequentially derived from a loss of *m/z* = 72 (-[C_2_H_4_OCO]) and 73 (-[C_2_H_5_OCO]). The *N*-ethoxycarbonylation derivatives of each class of diamines showed a unique fragmentation behavior. Hence, the knowledge of the fragmentation mechanisms could potentially be used to identify the structure of diamines that have no available authentic standards but have similar chemical structures to known diamines.

### 2.4. Validation of Analytical Parameters

The developed method was validated with parameters of linearity (internal standard calibration curve), sensitivity (limits of detection (LODs) and limits of quantification (LOQs)), stability, accuracy (extraction recovery), and precision (repeatability and reproducibility) under optimum conditions [30]. For linearity, the diamines standard solutions and internal standards were subjected to 2M HCl/acetonitrile (*v/v* = 1:1) for derivatization and extraction procedures described above (Section 3.4). The internal standard calibration curve (y = ax + b) was constructed by plotting the peak area ratio (y) against the concentration (x) of each diamine. To check the linearity, the correlation coefficient (*R*) was applied. Table 3 shows that good linearity was observed over the obtained concentration range, as indicated by correlation coefficient (*R*) > 0.9990. The LODs and LOQs which were determined by measuring dilutions of standards until the *S/N* was 3 and 10, ranged from 0.0025 to 0.057 µg/mL and from 0.0079 to 0.018 µg/mL. The LODs and LOQs were far below the requirement of BGV. These results showed that the developed method provide the necessary sensitivity. The stability of *N*-ethoxycarbonylation derivatives was assessed during storage for 0, 6, 12, 18, and 24 at room temperature. The RSD was less than 2.35% and the *N*-ethoxycarbonylation derivative were known to be stable for at least 24 h.

The extraction recovery was tested by spiking with low and high levels of diamine standards in PBAT mulch film samples. The spiked sample was incubated overnight at 4 °C and then extracted. The extraction recovery (%) was calculated using the equation, Recovery% = (C_spiked sample_ − C_sample_) × 100/C_spiked_, where C_spiked sample_ and C_sample_ are the diamines content in spiked and unspiked samples, respectively. C_spiked_ is the standard content. The recovery was between 93.9% and 101.2%, which indicated that the developed method exhibited high extraction efficiency (Table 4). Repeatabilities (within-day precision) were calculated by analyzing five independent samples within a single day, while reproducibilities (between-day precision) were calculated by analyzing five independent samples over three consecutive days. The repeatability and reproducibility varied from 0.89 to 8.12% and 2.12 to 10.56%, respectively. All these parameters, which are shown in Table 4, meet the requirement at acceptable ranges of International Council Harmonization (ICH) guideline [38].

### 2.5. Analysis of Residual Diisocyanates and Related Diamines in PBAT Mulch Film Samples

Our developed method was applied to commercial PBAT mulch film samples for analysis of diverse residual diisocyanates and related diamines. According to the results in Table 5, the components varied greatly among the samples from different origins. PBAT-A contained a high content of 2, 6-TDA and 2, 4-TDA but a lower content in PBAT-B. HDA was only found in PBAT C. Other diisocyanates and related diamines were not detected. The aromatic diisocyanates (2, 6-TDI and 2, 4-TDI), precursors of 2, 6-TDA and 2, 4-TDA were widely applied biodegradable polymers as functions for chain extender and compatibilizer [39]. The added diisocyanates can provide improvement in mechanical properties, good interfacial adhesion with polymer blends and show a homogeneous distribution on the surface [40]. Generally, 2, 6-TDI and 2, 4-TDI are the predominant diisocyanates in the global market, but have toxicity with acute or chronic effects and potential carcinogenicity. Safer aliphatic biocompatible diisocyanates (HDA and DAB) are suitable alternatives in recent years [41,42]. As a result, diisocyanates and related diamines need to be effectively analyzed for assessing the safety in different biodegradable mulch films.

## 3. Materials and Method

### 3.1. Chemicals and Reagent

All chemicals and reagents used in this study were of analytical grade unless otherwise stated. The diisocyanate standards of 1, 4-diisocyanatobutane (DICB, Lot. A0404071, Acros Organics), hexamethylene diisocyanate (HDI, Lot. F1914120, Aladdin), isophorone diisocyanate (IPDI, Lot. STBH9486, Sigma), L-lysine diisocyanate (LDI, Lot. C10660329, Macklin), toluene-2, 6-diisocyanate (2, 6-TDI, Lot. SHBF4477V, Sigma), toluene-2, 4-diisocyanate (2, 4-TDI, Lot. SHBJ9078, Sigma), and methylene bis(4-cyclohexylisocyanate) (HMDI, Lot. BCCB9581, Sigma), and diamine standards of 1, 4-diaminobutane (DAB, Lot. F1917182, Aladdin), 1,6-hexamethylenediamine (HDA, Lot. 20130509, Sinopharm), isophoronediamine (IPDA) (cis- and trans- mixture, Lot. G1816097, Aladdin), L-lysine ethyl ester dihydrochloride (LEE, Lot. G1816031, Aladdin), 2, 6-toluenediamine (2, 6-TDA, Lot. H1808124, Aladdin), 2, 4-toluenediamine (2, 4-TDA, Lot. A1911048, Aladdin), and 4, 4′-diaminodicyclohexylmethane (DDCM, Lot. LTC0S64, J&K) were used with purities ≥98%. The internal standard (IS) of 1, 5-diaminopentane (DAP, Lot. J2010080, Aladdin) and derivatization reagents of methyl chloroformate (MCF, Lot. 201907181, Aike), ethyl chloroformate (ECF, Lot. 201907151, Aike), propyl chloroformate (PCF, Lot. LAB0U31, J&K), isobutyl chloroformate (IBCF, Lot. UJZ0B-PC, TCI) were obtained with purities ≥98%. Deionized water was obtained from Milli-Q water purification system. The physical-chemical properties of the related diamines were presented in Table 2.

### 3.2. Preparation of Standard Solution and Biodegradable Mulch Film Samples

The mixed standard solution of the diisocyanates (DICB: 0.203 mg mL^−1^, HDI: 0.203 mg mL^−1^, IPDI: 0.225 mg mL^−1^, LDI: 0.203 mg mL^−1^, 2, 6-TDI: 0.228 mg mL^−1^, 2, 4-TDI: 0.204 mg mL^−1^, HMDI: 0.203 mg mL^−1^) were prepared in ethyl acetate. A mixed standard of related diamines and the IS solution were prepared at a final concentration of 0.2 mg mL^−1^ in 0.2 M HCl. All standards were stored in brown vials at 4 °C.

To investigate the component of diisocyanates and related diamines additives in the PBAT biodegradable mulch films from different manufacturers, three samples of PBAT-1, PBAT-2, and PBAT-3, which are widely used in agricultural production, were selected. Six repetitive samples were taken and then cut into small square-shaped pieces of ~1 mm. These samples were stored in glass bottles for further analysis.

### 3.3. Ultrasonic Hydrolysis and Extraction of Residual Diisocyanates and Related Diamines

PBAT biodegradable mulch films samples (50 mg) and the IS solution (10 μL) were placed in a 10 mL glass centrifuge tubes, followed by addition 3 mL of 2 M HCl/acetonitrile (*v/v* = 1:1). After mixing with vortex mixer for 1 min, the extraction was performed for 76 min at 30 °C with ultrasonic intensity at 100 W. The extraction solution was filtered through a 0.22 µm nylon membrane into a 10 mL glass centrifuge tubes for ECF derivatization.

### 3.4. N-Ethoxycarbonylation Derivatization of Diamines

The hydrolyzate was adjusted to pH = 7 with Na_2_CO_3_ solid, and then the reaction mixture was further adjusted to pH 11 with 2.5 M Na_2_CO_3_ (200 µL) solution. To carry out the *N*-ethoxycarbonylation derivatization of diamines and extraction of *N*-ethoxycarbonylation derivatives, 1 mL of ethyl acetate containing 50 µL of ECF was added and shaken for 1–2 min. After centrifuged 3 min at 3000 rpm, the organic layer containing the *N*-ethoxycarbonylation derivatives was transferred to a glass vial. This derivatization was repeated, and the combined ethyl acetate was evaporated to dryness under a stream of nitrogen. The derivatives were reconstituted with 100 µL of ethyl acetate and transferred into a 150 µL autosampler vial insert with polymer feet. A 1.0 µL aliquot was injected for GC-MS analysis.

### 3.5. Instruments and Chromatographic Conditions

An Agilent 7890A-GC system equipped with a 5975C-MS was used for the detection of the *N*-ethoxycarbonylation derivatives. Separation was performed with a DB-17 capillary column (30 m × 0.25 mm i.d. × 0.25 μm film thickness). The constant flow rate of helium carrier gas (99.999%) was maintained at 1 mL min^−1^. Injector temperature was maintained at 280 °C with a split ratio of 20:1. The initial temperature was 60 °C for 2 min, then ramped at 5 °C min^−1^ to 230 °C and held for 3 min, followed by 15 °C min^−1^ to 280 °C and held for 10 min with a total run time of 52.33 min. The ion source and mass quadrupole temperatures were 230 °C and 150 °C, respectively. The transfer line temperature and solvent delay were set to 280 °C and 25 min. All mass spectra were acquired in electron impact (EI) at 70 eV with full scan (*m/z* range, 50–600) for identification and selected ion monitoring (SIM) for quantification. The retention index (RI) was calculated using retention times associated with *n*-paraffins (C6-C40) and AMDIS software (Automated Mass Spectral Deconvolution and Identification System). The retention time, RI, and characteristic ions of corresponding *N*-ethoxycarbonylation derivative of diamines were presented in Table 2.

### 3.6. Statistical Analysis

The response surface methodology of the BBD was used to optimize the hydrolysis efficiency of ultrasonic hydrolysis and extraction, and the ANOVA of BBD was conducted using the Design-Expert version 8.0.6 software (Stat-Ease Inc., Minneapolis, MN, USA). To measure how well the proposed model fit the experimental data, the parameters, such as model *p*-value, lack of fit, and CV and *R*^2^, were used for evaluation. The model *p*-value and CV less than 0.05 and 10%, lack of fit, and *R*^2^ greater than 0.05 and 0.90 showed a reliable model was obtained. The Student’s *t*-test and ANOVA with Fisher’s least significant difference (LSD) test were applied for statistical comparison with *p* < 0.05 among different conditions using SPSS 16.0 software (SPSS Inc., Chicago, IL, USA). All diagrams were drawn using Origin 2021 Software (Origin Lab Corp., Northampton, MA, USA) and ChemBioDraw Ultra 12.0 (Cambridgesoft.com, Cambridge, MA, USA).

## 4. Conclusions

This study presents an ultrasonic hydrolysis and extraction, ECF derivatization followed GC-MS method for identification and quantitation of residual diisocyanates and related diamines in PBAT biodegradable mulch films. The ultrasonic acid hydrolysis extraction was optimized with BBD and degradation of diamines in aqueous phase under alkaline conditions were evaluated at *N*-ethoxycarbonylation conditions. Through careful optimization, the method exhibited high selectivity, lower LODs, broad linear ranges, adequate accuracy, and high precision. The *N*-ethoxycarbonylation derivatives of each class of diamines showed a unique fragmentation behavior. These fragmentation mechanisms can be used for identification of unknown diamines. The developed method provides a feasible approach for determination of diverse residual diisocyanates and related diamines.

## Figures and Tables

**Table 1:** BBD of uncoded (coded) variables, average hydrolysis efficiency of each class diisocyanates and model coefficient of ANOVA.

	Uncoded (Coded) Variables				Average Hydrolysis Efficiency of Each Class Diisocyanates			
No	A/*v/w*	B/W	C/°C	D/min	Aliphatic	Cycloaliphatic	Amino Acid Ester	Aromatic
1	60 (0)	300 (1)	50 (0)	30 (−1)	0.758	0.765	0.695	0.643
2	60 (0)	200 (0)	50 (0)	60 (0)	0.848	0.855	0.795	0.732
3	60 (0)	300 (1)	50 (0)	90 (1)	0.854	0.865	0.823	0.746
4	40 (−1)	200 (0)	70 (1)	60 (0)	0.911	0.923	0.652	0.795
5	80 (1)	100 (−1)	50 (0)	60 (0)	0.945	0.956	0.885	0.822
6	80 (1)	200 (0)	70 (1)	60 (0)	0.932	0.941	0.692	0.805
7	40 (−1)	100 (−1)	50 (0)	60 (0)	0.921	0.935	0.855	0.792
8	60 (0)	200 (0)	50 (0)	60 (0)	0.885	0.891	0.791	0.761
9	40 (−1)	300 (1)	50 (0)	60 (0)	0.862	0.885	0.785	0.751
10	60 (0)	300 (1)	30 (−1)	60 (0)	0.885	0.902	0.812	0.755
11	80 (1)	200 (0)	50 (0)	90 (1)	0.928	0.914	0.845	0.777
12	60 (0)	300 (1)	70 (1)	60 (0)	0.882	0.896	0.675	0.764
13	80 (1)	200 (0)	30 (−1)	60 (0)	0.905	0.912	0.821	0.812
14	60 (0)	200 (0)	50 (0)	60 (0)	0.865	0.885	0.818	0.752
15	60 (0)	100 (−1)	50 (0)	30 (−1)	0.865	0.883	0.754	0.761
16	60 (0)	200 (0)	50 (0)	60 (0)	0.892	0.912	0.789	0.785
17	40 (−1)	200 (0)	30 (−1)	60 (0)	0.918	0.932	0.849	0.798
18	80 (1)	200 (0)	50 (0)	30 (−1)	0.801	0.825	0.662	0.711
19	60 (0)	100 (−1)	50 (0)	90 (1)	0.932	0.918	0.835	0.794
20	60 (0)	100 (−1)	70 (1)	60 (0)	0.952	0.934	0.701	0.811
21	40 (−1)	200 (0)	50 (0)	90 (1)	0.912	0.915	0.823	0.799
22	60 (0)	200 (0)	30 (−1)	90 (1)	0.885	0.899	0.785	0.765
23	40 (−1)	200 (0)	50 (0)	30 (−1)	0.792	0.784	0.652	0.671
24	60 (0)	100 (−1)	30 (−1)	60 (0)	0.915	0.922	0.829	0.794
25	60 (0)	200 (0)	30 (−1)	30 (−1)	0.762	0.766	0.652	0.645
26	60 (0)	200 (0)	50 (0)	60 (0)	0.871	0.896	0.828	0.752
27	60 (0)	200 (0)	50 (0)	60 (0)	0.852	0.882	0.811	0.745
28	60 (0)	200 (0)	70 (1)	90 (1)	0.871	0.881	0.628	0.761
29	60 (0)	200 (0)	70 (1)	30 (−1)	0.785	0.795	0.605	0.682
30	80 (1)	300 (1)	50 (0)	60 (0)	0.835	0.845	0.802	0.722
ANOVA		Model *p*-value			<0.0001	<0.0001	<0.0001	<0.0001
		Lack of fit *p*-value			0.2638	0.3110	0.0712	0.3418
		Coefficient of variation %			2.49	2.53	3.61	2.72
		*R* ^2^			0.9115	0.9018	0.9385	0.9034

**Table 2:** Physical-chemical properties of the related diamines; retention time, retention index, and characteristic ions of *N*-ethoxycarbonyl derivative of diamines.

		Diamines			*N*-Ethoxycarbonyl Derivative of Diamines		
Diamines	Structure	Class	pK_a_	Log K_ow_	RT ^a^/min	RI ^b^	Characteristic Ions ^c^/*m/z*
DAB	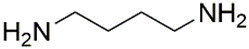	Aliphatic diamine	10.51	−0.69	25.487	1863.8	102; *142*; 159; 187; **232**
DAP (IS)	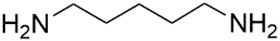	Aliphatic diamine	10.51	−0.16	27.681	1974.3	102; *156*; 128; 201; **246**
HDA	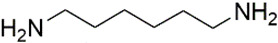	Aliphatic diamine	10.51	0.04	29.562	2077.1	102; *130*; 158; 215; **260**
IPDA	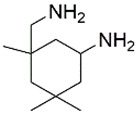	Cycloaliphatic diamine	10.54	0.96	30.472	2185.7/ 2264.1	123; *212*; 241; 269; **314**
LEE	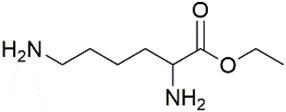	Amino acid ester	7.33	—	32.374	2264.1	156; 128; *226*; 171; 272; **318**
2, 6 -TDA	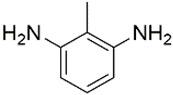	Aromatic diamine	8.42	2.34	39.626	2401.1	**266**; 121; *147*; 194; 220
2, 4 -TDA	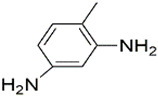	Aromatic diamine	9.31	1.99	40.239	2360.2	**266**; *121*; 147; 194; 220
DDCM	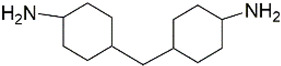	Cycloaliphatic diamine	10.87	1.59	42.482	2384.5	128; *184*; 265; 309; **354**

^a^ Retention time; ^b^ Retention index; ^c^ The *m/z* in underline and italic is quantitative and confirmative ion for SIM, respectively. The *m/z* in bold is molecular ion.

**Table 3:** Linearity, LOD, LOQ and stability of *N*-ethoxycarbonylation derivatives with the developed method.

Diamines	Linear Range/µg/mL	Regression Equation	*R*	LOD/µg/mL	LOQ/µg/mL	Stability
DAB	0.02~2.00	y = 0.385x + 0.0146	0.9999	0.0043	0.013	0.66
HDA	0.04~4.00	y = 0.315x + 0.0220	0.9997	0.0051	0.016	0.76
IPDA	0.02~2.00	y = 0.560x + 0.0265	0.9995	0.0025	0.0079	0.81
LEE	0.02~2.00	y = 0.359x − 0.00458	0.9992	0.0047	0.015	1.09
2, 6-TDA	0.04~4.00	y = 0.441x − 0.00637	0.9996	0.0036	0.011	2.35
2, 4-TDA	0.04~4.00	y = 0.487x + 0.00311	0.9996	0.0031	0.0097	1.57
DDCM	0.02~2.00	y = 0.269x + 0.00950	0.9990	0.0057	0.018	1.03

**Table 4:** Recovery, repeatability, and reproducibility of *N*-ethoxycarbonylation derivatives with the developed method.

Diamines	Content/ µg/g	Concentration/µg/g		Recovery (Repeatability)/%		Reproducibility/%
		Low Spiked	High Spiked	Low Spiked	High Spiked	
DAB	—	0.10	1.00	98.3 (1.25)	99.6 (0.89)	2.12
HDA	—	5.00	10.00	97.2 (2.00)	101.2 (1.45)	3.08
IPDA	—	0.10	1.00	97.6 (2.44)	99.7 (2.02)	2.98
LEE	—	0.10	1.00	95.5 (4.89)	98.2 (3.18)	6.12
2, 6-TDA	21.85	10.00	20.00	94.7 (6.74)	97.7 (4.65)	8.34
2, 4-TDA	63.81	30.00	60.00	93.9 (8.12)	98.1 (6.24)	10.56
DDCM	—	0.10	1.00	95.3 (3.45)	99.3 (2.77)	5.11

**Table 5:** The content of diisocyanates and related diamines in three PBAT biodegradable mulch film samples.

Content/µg/g	PBAT-1	PBAT-2	PBAT-3
DAB	ND ^a^	ND	ND
HDA	ND	ND	10.23 ± 0.28
IPDA	ND	ND	ND
LEE	ND	ND	ND
2, 6-TDA	0.69 ± 0.08	21.85 ± 1.64	ND
2, 4-TDA	1.43 ± 0.15	63.81 ± 4.05	ND
DDCM	ND	ND	ND

^a^ ND: The content is lower than LOD.

## Data Availability

Not applicable.

## Supplementary Materials

The following supporting information can be downloaded at: https://www.mdpi.com/article/10.3390/molecules27196754/s1, Table S1: Optimization of ECF volume for N-ethoxycarbonylation derivatization; Table S2: Optimization of derivatization-extraction time for N-ethoxycarbonylation derivatization; Table S3: Optimization of extraction solvent for N-ethoxycarbonylation derivatization.
