# Supplementary material for: Determination of Residual Diisocyanates and Related Diamines in Biodegradable Mulch Films Using N-Ethoxycarbonylation Derivatization and GC-MS
Source: Molecules. 2022 Oct 10;27(19):6754. doi: 10.3390/molecules27196754 (PMC9572079; doi:10.3390/molecules27196754)
Supplement: Supplementary file 1 [file molecules-27-06754-s001.zip › molecules-1967774-supplementary.pdf]

Table S1 Optimization of ECF volume for *N*-ethoxycarbonylation derivatization

Diamines	ECF volume / Relative response factors as derivatization efficiency [a]				
	10 μL	30 μL	50 μL	70 μL	90 μL
DAB	0.61a ± 0.02	0.79b±0.02	0.80b±0.02	0.81b±0.02	0.75b±0.03
HDA	0.92a±0.05	1.15b±0.04	1.20b±0.04	1.21b±0.05	1.17b±0.05
IPDA	0.58a±0.01	0.76b±0.02	0.78b±0.02	0.77b±0.02	0.71b±0.02
LEE	0.55a±0.02	0.74b±0.02	0.77b±0.02	0.78b±0.03	0.69b±0.03
2,6-TDA	0.48a±0.03	0.60b±0.02	0.62b±0.03	0.60b±0.03	0.58b±0.03
2,4-TDA	0.75a±0.05	0.91b±0.03	0.91b±0.04	0.92b±0.04	0.87b±0.05
DDCM	0.55a±0.02	0.79b±0.02	0.80b±0.02	0.81b±0.03	0.75b±0.03

[a] The relative response factors were the peak area of the *N*-ethoxycarbonylation derivatives of diamines divided by the internal standard (DAP).

Table S2 Optimization of derivatization-extraction time for *N*-ethoxycarbonylation derivatization

Diamines	Derivatization-extraction time / Relative response factors as derivatization efficiency [a]				
	1 min	2 min	3 min	4 min	5 min
DAB	0.82a ±0.03	0.84a ±0.03	0.83a ±0.04	0.80a ±0.02	0.85a ±0.04
HDA	1.15a ±0.04	1.19a ±0.04	1.25a ±0.05	1.20a ±0.04	1.25a ±0.05
IPDA	0.80a ±0.03	0.82a ±0.02	0.83a ±0.02	0.83a ±0.03	0.84a ±0.02
LEE	0.73a ±0.02	0.76a ±0.02	0.77a ±0.03	0.78a ±0.02	0.78a ±0.03
2,6-TDA	0.54a ±0.04	0.59a ±0.04	0.57a ±0.05	0.57a ±0.04	0.58a ±0.05
2,4-TDA	0.91a ±0.06	0.96a ±0.04	0.93a ±0.05	0.94a ±0.06	0.94a ±0.05
DDCM	0.86a ±0.03	0.85a ±0.04	0.83a ±0.04	0.84a ±0.03	0.84a ±0.03

[a] The relative response factors were the peak area of the *N*-ethoxycarbonylation derivatives of diamines divided by the internal standard (DAP).

Table S3 Optimization of extraction solvent for *N*-ethoxycarbonylation derivatization

Diamines	Different polarity solvent / Relative response factors as derivatization efficiency [a]					
	Petroleum ether	*n*-Hexane	Methyl tert-butyl ether	Ethyl ether	Ethyl acetate	Chloroform
DAB	0.04a ±0.004	0.08b ±0.008	0.80b ±0.04	0.83b ±0.05	0.85b ±0.03	0.86b ±0.06
HDA	0.79a ±0.06	0.97b ±0.07	1.24c ±0.05	1.24c ±0.04	1.27c ±0.04	1.25c ±0.05
IPDA	0.18a ±0.01	0.17a ±0.01	0.72b ±0.03	0.79b ±0.03	0.80b ±0.04	0.82b ±0.04
LEE	0.23a ±0.03	0.23a ±0.02	0.77b ±0.04	0.81b ±0.03	0.83b ±0.03	0.83b ±0.04
2,6-TDA	0.42a ±0.10	0.47a ±0.08	0.48a ±0.06	0.60b ±0.04	0.60b ±0.05	0.62b ±0.06
2,4-TDA	0.54a ±0.15	0.63a ±0.12	0.84b ±0.06	0.91b ±0.05	0.93b ±0.05	0.95b ±0.08
DDCM	0.04a ±0.01	0.08a ±0.02	0.78b ±0.04	0.83b ±0.05	0.85b ±0.04	0.84b ±0.07

[a] The relative response factors were the peak area of the *N*-ethoxycarbonylation derivatives of diamines divided by the internal standard (DAP).
